# Mechanistic Foundations of KRAS‐Driven Tumor Ecosystems: Integrating Crosstalk among Immune, Metabolic, Microbial, and Stromal Microenvironment

**DOI:** 10.1002/advs.202502714

**Published:** 2025-06-09

**Authors:** Jiayao Ma, Shenao Fu, Jun Tan, Ying Han, Yihong Chen, Xiangying Deng, Hong Shen, Shan Zeng, Yinghui Peng, Changjing Cai

**Affiliations:** ^1^ Department of Oncology Xiangya Hospital Central South University Changsha Hunan 410008 China; ^2^ Department of Neurosurgery Xiangya Hospital Central South University Changsha Hunan 410008 China; ^3^ National Clinical Research Center for Geriatric Disorders Xiangya Hospital Central South University Changsha Hunan 410008 China

**Keywords:** cancer, immunity, KRAS, metabolism, microbiota, microenvironment, stroma

## Abstract

Kirsten rat sarcoma viral oncogene homolog (KRAS) is the most frequently mutated member of the RAS family of small GTPases (RAS). It affects about one‐fifth of cancer cases. The tumor microenvironment (TME) is a multifaceted network of immune cells, metabolites, microbiota, stromal components, and extracellular matrix. It creates a dynamic ecosystem that supports malignant initiation, progression, and therapy resistance through bidirectional crosstalk with tumor cells. Emerging evidence reveals distinct TME landscapes shaped by wild‐type versus oncogenic KRAS variants. Additionally, TME rewiring occurs during KRAS‐targeted therapies. Deciphering these KRAS‐dependent TME architectures and their therapeutic vulnerabilities represents a critical frontier for precision oncology. This review synthesizes key milestones and persistent challenges in KRAS inhibitor development. And it systematically evaluates how KRAS mutations orchestrated immunosuppressive niches, metabolic symbiosis, stromal remodeling, and microbiome dysbiosis, supported by mechanistic insights from preclinical and clinical studies. It further explores therapeutic opportunities arising from targeting TME interactions, including rational combinations of KRAS inhibitors with immune checkpoint blockade, metabolic agents, or microbiota‐modulating strategies.

## Graphical abstract

KRAS mutations dynamically remodel the tumor microenvironment (TME), fostering immunosuppression, metabolic symbiosis, and stromal plasticity. This review dissects how oncogenic KRAS variants sculpt TME networks and explores actionable vulnerabilities. Emerging strategies combining KRAS inhibitors and TME‐editing approaches offer potential to dismantle tumor‐ecosystem crosstalk and advance precision oncology.

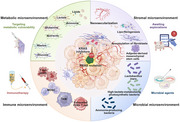

## 1 Background

**Figure 1:**
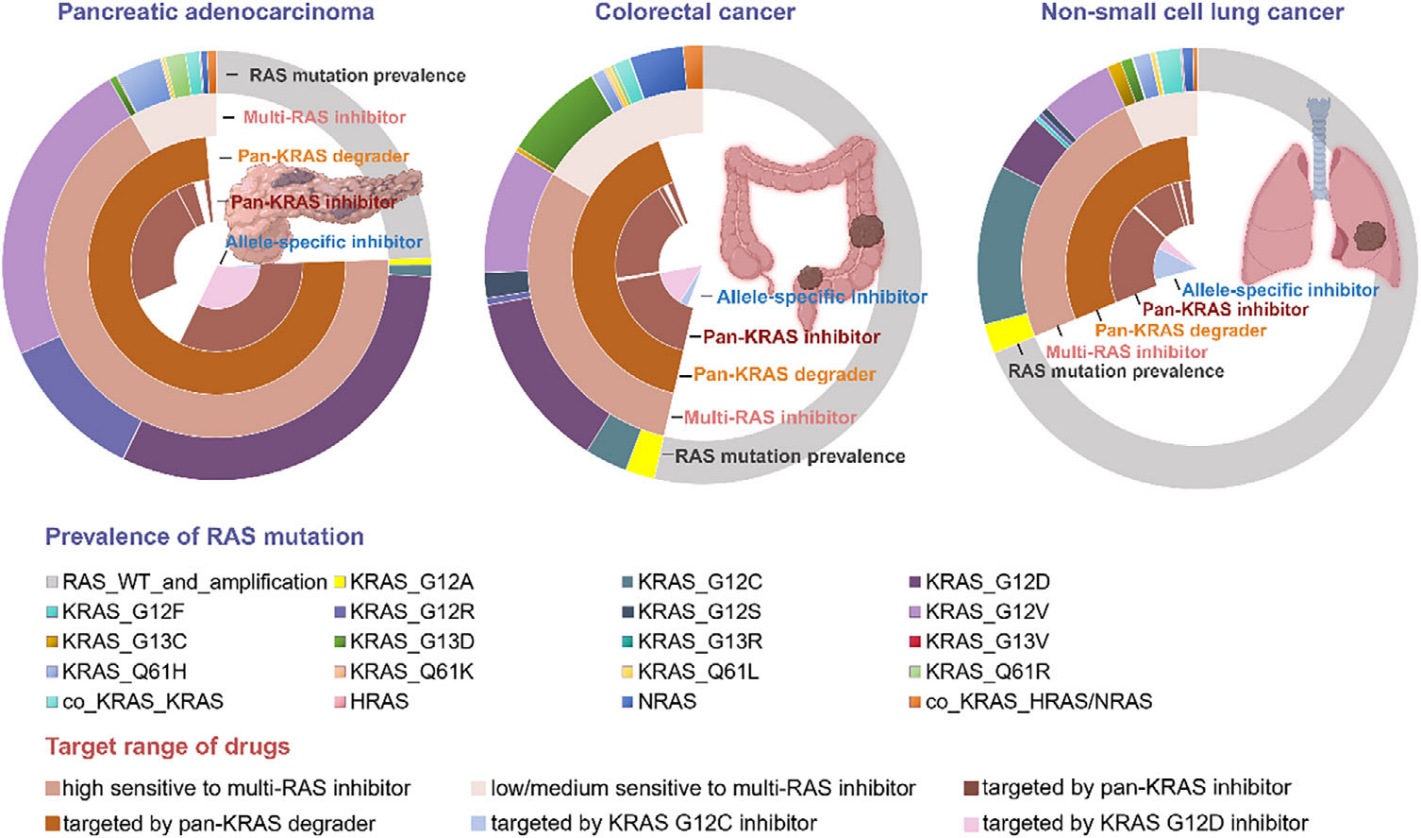
The prevalence of RAS mutations and the target range of KRAS/RAS inhibitors/degraders.

Mutations in the RAS family of small GTPases occur in approximately one‐fifth of all human cancers.^[^ ^1^ ^]^ Kirsten rat sarcoma viral oncogene homolog (KRAS) is the most frequently mutated member, with an especially high mutation rate in pancreatic adenocarcinoma (PDAC), and a notable incidence in appendiceal adenocarcinoma, small bowel adenocarcinoma, colorectal cancer (CRC), and nonsmall cell lung cancer (NSCLC) (**Figure** **1**).^[^ ^2^ ^]^ This underscores the significant therapeutic demand for effective KRAS inhibitors.

This figure visualized the percentages of cases with various RAS mutations in pancreatic adenocarcinoma, colorectal cancer, and nonsmall cell lung cancer. The target ranges of KRAS G12C/D inhibitors, pan‐KRAS inhibitors/degraders, and multi‐RAS inhibitors were also presented.

The wild‐type KRAS gene encodes a GTPase protein which functions as a molecular switch. The inherently slow GTP hydrolysis rate in KRAS could be catalytically accelerated by GTPase‐activating proteins (GAPs), switching KRAS to its inactive state.^[^ ^3^ ^]^ Conversely, guanine nucleotide exchange factors (GEFs) facilitate the nucleotide exchange in KRAS, where GDP is released to enable GTP binding, and thereby activating KRAS to interact with effector proteins.^[^ ^4^ ^]^ Normally, this cycling between active and inactive states is tightly regulated by GAPs and GEFs.^[^ ^5^ ^]^ Mutations in KRAS often elevate the levels of active KRAS, thereby driving PI3K‐AKT and MEK‐ERK signaling pathways.^[^ ^3^, ^6^ ^]^ Such signaling leads to uncontrolled cell proliferation and tissue deformation, culminating in tumorigenesis and progression.^[^ ^7^ ^]^ Most KRAS mutations involve base substitutions in codons 12, 13, or 61,^[^ ^2^ ^]^ with G12C (Glycine to Cysteine), G12D (Glycine to Aspartic acid), and G12V (Glycine to Valine) as hotspots (Figure 1).^[^ ^2b^ ^]^ These variants display distinct conformations and biological behaviors, posing substantial challenges for drug development.^[^ ^6^, ^8^ ^]^ Although allele‐specific inhibitors have partially met the therapeutic needs for tumors harboring KRAS G12C or G12D mutations,^[^ ^9^ ^]^ treatment options remain inadequate for patients with other oncogenic KRAS alleles or comutations. Since 2023, a new wave of promising drugs targeting multiple KRAS alleles has emerged. These agents are categorized into three groups: pan‐KRAS inhibitors that target wild‐type and most oncogenic KRAS alleles for suppression;^[^ ^10^ ^]^ pan‐KRAS degraders that target wild‐type and most oncogenic KRAS alleles for degradation;^[^ ^11^ ^]^ and multi‐RAS inhibitors that target wild‐type and multiple oncogenic alleles of KRAS, HRAS, and NRAS for suppression (Figure 1).^[^ ^12^ ^]^ These advances herald a new era when multiple KRAS variants can be targeted with one strategy, referred to in this article as “the multi‐KRAS era.”

The tumor microenvironment (TME) consists of a complex network of immune cells, metabolites, microorganisms, stromal cells, and the extracellular matrix, forming the milieu where tumor cells thrive. The TME can be categorized into immune, metabolic, microbial, and stromal subtypes.^[^ ^13^ ^]^ These components engage in continuous cross‐talk and coevolved with tumor cells, significantly influencing drug response, toxicity, and resistance.^[^ ^13^, ^14^ ^]^ There are both similarities and differences in the TMEs of tumors with wild‐type KRAS and those with KRAS mutations.^[^ ^15^ ^]^ Comprehending the TME landscapes associated with KRAS variants and their inhibitors is crucial for precise TME regulation, representing the next frontier in the multi‐KRAS era.

This review article highlighted the milestones achieved and the challenges in the development of KRAS inhibitors. It proceeded to examine the characteristics of the TME related to KRAS mutations and inhibitors, with dedicated sections analyzing immunity, metabolism, the extracellular matrix, and microbiota. Furthermore, the review delved into the interconnected network among immune, metabolic, stromal, and microbial components. It concluded with a discussion on the targeting strategies of the TME, emphasizing their potential to combine with KRAS inhibitors and degraders. The TME landscape and related targeting strategies in KRAS mutant tumors represent explosive research areas that deserve further investigation.

## 2 The Milestones and Dilemmas in the Development of KRAS Inhibitors

**Figure 2:**
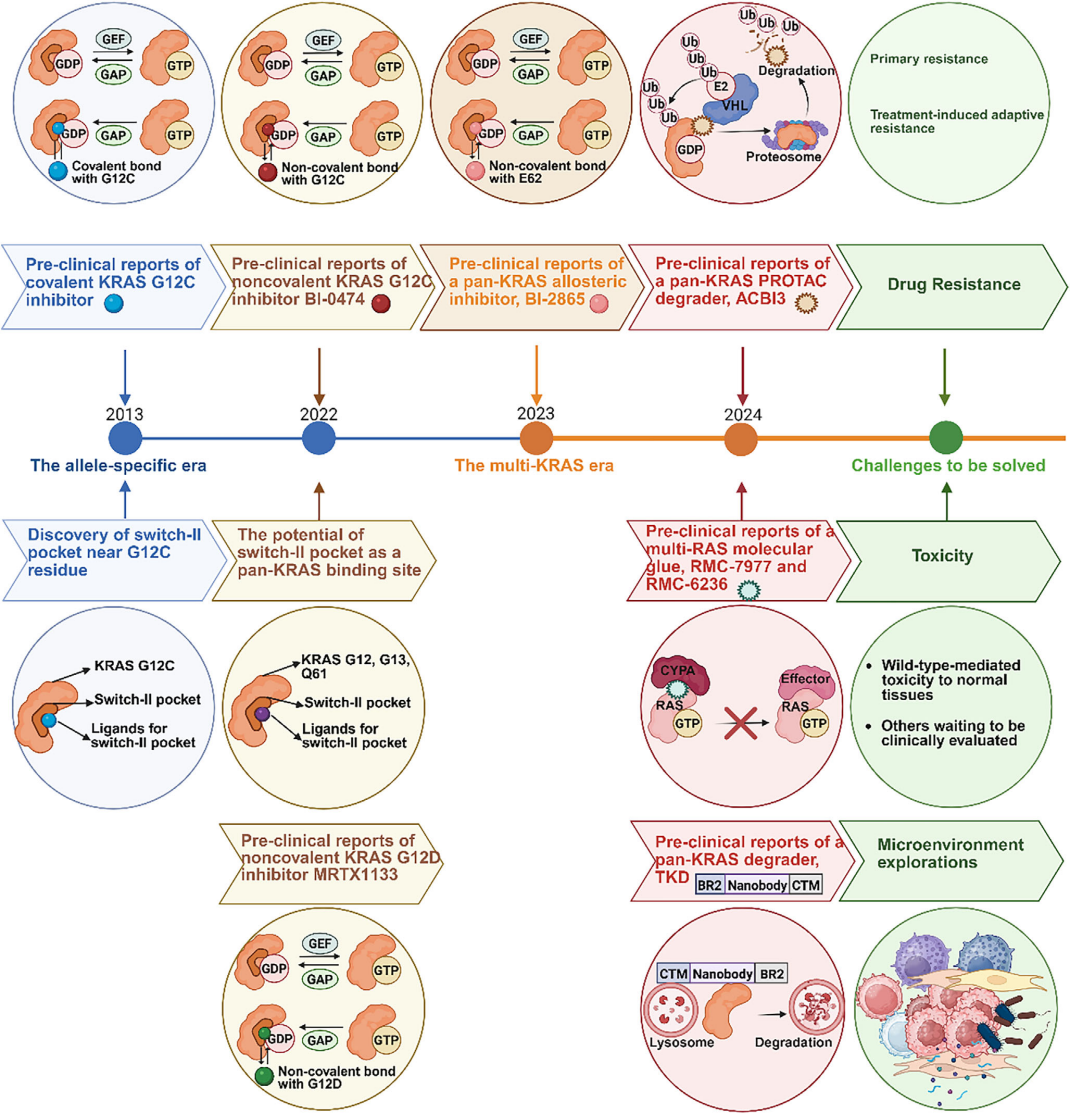
The milestones achieved in KRAS drug development and ongoing challenges in the development of KRAS inhibitors.

The milestones and dilemmas in the development of KRAS inhibitors are summarized in **Figure** **2**. KRAS was long deemed undruggable due to the absence of identifiable druggable pockets. A significant breakthrough occurred in 2013 with the identification of the switch‐II pocket, a novel allosteric site beneath the effector‐binding switch‐II region in KRAS G12C. Small molecular inhibitors that covalently bound with G12C residue and irreversibly embed into the switch‐II pocket were developed.^[^ ^9a^ ^]^ These inhibitors block KRAS G12C activation by interrupting the nucleotide exchange. And two of them, sotorasib and adagrasib, have got accelerated approvals by FDA for clinical use in adult patients with KRAS G12C‐mutated locally advanced or metastatic NSCLC who have received at least one prior systemic therapy (thus second‐ or later‐line therapies), based on phase 1/2 clinical trials CodeBreaK 100^[^ ^16^ ^]^ and KRYSTAL‐1,^[^ ^17^ ^]^ respectively. It marks the beginning of KRAS allele‐specific therapeutics. Additionally, Phase 3 clinical trials of these drugs, as well as trials targeting a broader range of tumor types, are ongoing. Clinical evaluations of sotorasib in combination with other drugs as a first‐line treatment are also underway (CTR20240724).^[^ ^18^ ^]^ Other KRAS G12C inhibitors, such as divarasib (NCT06497556)^[^ ^19^ ^]^ and garsorasib(NCT06300177),^[^ ^20^ ^]^ are also being tested in Phase 3 trials. Undoubtedly, KRAS G12C inhibitors are rapidly advancing and hold promising prospects for widespread clinical application. However, these inhibitors rely on the covalent bonds with the cysteine residue to work, which restricts their efficacy on KRAS mutants besides KRAS G12C. Treatment options remained inadequate for patients with other oncogenic KRAS alleles or comutations in the era of KRAS allele‐specific therapeutics.

The beginning of the KRAS allele‐specific era was marked by preclinical reports of the KRAS G12C inhibitor in 2013. And the advent of the multi‐KRAS era was signified by the report of the first pan‐KRAS inhibitor, BI‐2865, in 2023. Challenges that persist in the multi‐KRAS era include drug resistance, toxicity, and unclear interactions with the tumor microenvironment.

In 2022, MRTX1133 and BI‐0474, small molecules that noncovalently and reversibly bind to the switch II pocket in KRAS G12D and G12C respectively, were reported,^[^ ^9c,d^ ^]^ providing a starting point for the development of noncovalent KRAS inhibitors. In the same year, researchers showed that the switch‐II pocket is accessible across various KRAS hotspot mutants (G12, G13, Q61) through noncovalent and reversible bonds, underscoring its potential as a druggable site for multiple KRAS variants.^[^ ^21^ ^]^ In the following year, pan‐KRAS inhibitors, BI‐2865 and BI‐2493, were reported.^[^ ^10^ ^]^ BI‐2865 was developed from BI‐0474 by removing the G12C covalent warhead. The cocrystal structures analysis of BI‐2865 and KRAS revealed a conserved binding pocket across KRAS variants which were defined by the α2 and α3 helices, the distal portion of the β1 sheet and the distal portion of the switch II motif in KRAS. In detail, BI‐2865 formed a direct ionic interaction with E62 in the switch II motif and a water‐mediated hydrogen bond network with the side chain of R68 and the main chain carbonyl of Q61. By binding to the off‐state KRAS and interrupting their nucleotide exchange and activation, BI‐2865 affected wild‐type KRAS and 15 KRAS mutants, including G12A/C/D/F/V/S, G13C/D, V14I, L19F, Q22K, D33E, Q61H, K117N, and A146V/T,^[^ ^10^ ^]^ representing a pivotal milestone in the multi‐KRAS era. BI‐2493 was an optimized form for in vivo administration. The more potent analog, BI‐3706674, has entered clinical trial (NCT06056024).

Targeted protein degradation technologies have also propelled the progress toward the multi‐KRAS era. Such drugs exploit the intracellular protein degradation system to eliminate pathogenic proteins.^[^ ^22^ ^]^ Protein degradation systems are classified into two categories: ubiquitin‐proteasome degradation and lysosomal degradation. Representative compounds harnessing the former one are PROteolysis TArgeting Chimeras (PROTACs).^[^ ^23^ ^]^ PROTACs comprise three components: a ligand for the target protein KRAS, a linker, and a ligand for recruiting E3 ubiquitin ligase. E3 ubiquitin ligases are enzymes that catalyze the transfer of ubiquitin from an E2 enzyme to specific substrate proteins. The von Hippel–Lindau (VHL) protein is a specific E3 ligase often used in PROTACs. PROTACs link the E3 ubiquitin ligase and KRAS together to form a ternary complex, facilitating the ubiquitination and subsequent proteasome degradation of KRAS.^[^ ^24^ ^]^ Early in 2021, BOND et al. reported LC2, a specific degrader recruiting VHL to induce the degradation of KRAS G12C.^[^ ^25^ ^]^ Two years later, Anthony et al. introduced ASP3082, a specific degrader targeting KRAS G12D by recruiting an undisclosed E3 ligase.^[^ ^26^ ^]^ In 2024, Popow et al. performed cocrystal structure analysis on BI‐2865 and its analogs, and identified a solvent‐exposed subpocket formed by the amino acids H95, E62, and D92 in KRAS.^[^ ^11b^ ^]^ They considered this pocket as a promising position to for PROTACs design. Using motif for this pocket, together with linkers and VHL ligase binders, they produced a pan‐KRAS degrader named ACBI3. ACBI3 degraded and inactivated 13 of the 17 most common KRAS mutants (including G12D and G12V) and wild‐type KRAS while sparing HRAS and NRAS.^[^ ^11b^ ^]^ More recently, pan‐KRAS degraders engaging the lysosomal degradation pathway has also gained achievement, represented by the tumor‐targeting KRAS degrader (TKD). TKD comprised three components: the cancer cell‐penetrating peptide BR2, a nanobody binding to KRAS, and a lysosomal recognition and binding motif CTM.^[^ ^11c^ ^]^ BR2 enabled the targeted recognition of tumor cells,^[^ ^27^ ^]^ while CTM promoted the recognition, binding, and degradation of KRAS proteins by the lysosome.^[^ ^28^ ^]^ The nanobody, with a significantly smaller molecular weight than conventional antibodies, enhanced tissue penetration and KRAS targeting. And this made it an ideal platform for developing pan‐KRAS degraders.^[^ ^29^ ^]^

Another milestone came with molecular glues. Certain natural products with a high affinity for cyclophilin A (CYPA), when appropriately modified, can serve as molecular glues that recruit CYPA to the active state of RAS.^[^ ^30^ ^]^ Molecular glues inactivate mutant RAS by dual mechanisms: preventing the interactions between RAS and its effectors; and modulating the residues in the switch II motif of RAS to facilitate GTP hydrolysis.^[^ ^31^ ^]^ Molecular glues specific for KRAS G12C were reported in 2023.^[^ ^32^ ^]^ In the subsequent year, multi‐RAS inhibitors, RMC‐7977 and RMC‐6236, were introduced. These inhibitors demonstrated efficacy against wild‐type RAS and various isoforms including KRAS, NRAS, and HRAS.^[^ ^12^ ^]^ The most significant and durable responses were observed in cell lines with KRAS G12X mutations, and moderate responses were noted in cell lines with other hotspot mutations like KRAS G13X, Q61H, and K117N.^[^ ^12b^ ^]^

Several challenges persist in the multi‐KRAS era. The first one is how to prevent and overcome drug resistance. Primary resistance exists in cancer cells that are not addicted to oncogenic KRAS signals.^[^ ^11^, ^12^ ^]^ For pan‐KRAS inhibitors working by interrupting the nucleotide exchange, primary resistance exists in tumors driven by KRAS G12R and Q61R/K/L.^[^ ^10^ ^]^ These mutants are activated by the damaged GTP hydrolysis instead of the increased nucleotide exchange. For multi‐RAS inhibitors based on CYPA and the pan‐KRAS degraders based on E3 ligase, primary resistance can be caused by the lack of intracellular CYPA and E3 ligase, respectively.^[^ ^11^, ^12^ ^]^ Adaptive resistance occurs under the treatment of specific KRAS inhibitors. Continuous treatment with KRAS G12C inhibitors could result in copy‐number gain of the KRAS G12C allele and positive selection of resistant KRAS mutations, leading to tumor rebound.^[^ ^33^ ^]^ Pan‐KRAS degraders and multi‐RAS inhibitors will likely be susceptible to resistance mechanisms that cause reactivation of ERK signaling.^[^ ^11^, ^12^ ^]^ However, the bedside data for adaptive resistance after multi‐RAS inhibition or pan‐KRAS degradation are currently scare and pending further disclosure.

Drug toxicity is an important issue for pan‐KRAS drugs. Since pan‐KRAS and multi‐RAS inhibitors can target wild‐type KRAS and RAS protein respectively, wild‐type‐mediated toxicity to normal tissues is a concern. Previous researches showed that the genetic ablation of KRAS in young mice led to the decreased activity in 8 months old, and the genetic ablation of all RAS in mice resulted in death within several weeks.^[^ ^34^ ^]^ This raised concerns about the safety of pan‐KRAS and multi‐RAS inhibitors.^[^ ^35^ ^]^ Recent data showed that in preclinical models, RMC‐7977 and RMC‐6236 are tumor‐selective, inducing substantial apoptosis and sustained inhibition of proliferation exclusively in tumor tissues while sparing normal tissues.^[^ ^12^, ^36^ ^]^ Similar situation occurred in the test of BI‐2865.^[^ ^10^ ^]^ Possible reasons for tolerance in normal tissues were the relatively low levels of active RAS‐GTP and the homeostatic mechanisms restoring equilibrium following the therapeutic pressure that existed in normal cells.^[^ ^37^ ^]^ Clinical data are needed to further evaluate the safety of pan‐KRAS drugs. RMC‐6236 represents the most advanced multi‐RAS inhibitor in current clinical trials. A summary of ongoing trials and patents involving multi‐KRAS inhibitors is provided in **Tables** **1** and **2**, respectively.

**Table 1:** Clinical trials of new drugs/vaccines targeting multi‐/pan‐KRAS.

New drug/vaccine	Target	Tumor type	NCT number	Phase	Monotherapy/combination	Sponsor	Status	Refs.
BI 3706674	Multiple mutant and wild‐type KRAS	Solid tumors	NCT06056024	Phase 1	Monotherapy	Boehringer ingelheim	Recruiting	[109]
PF‐07934040	Multi‐KRAS	NSCLC, CRC, PDAC^a)^	NCT06447662	Phase 1	Monotherapy/combined with Gemcitabine/ Nab‐paclitaxel/ Cetuximab/ Fluorouracil/ Oxaliplatin/ Leucovorin/ Bevacizumab/ Pembrolizumab/ Pemetrexed/ Cisplatin/ Paclitaxel/ Carboplatin	Pfizer	Recruiting	/
RMC‐6236	KRAS G12X	Solid tumors	NCT06162221	Phase 1/2	Combined with Pembrolizumab, with or without chemotherapy	Revolution Medicines, Inc.	Recruiting	[110]
RMC‐6236	KRAS G12X	CRC, PDAC	NCT06445062	Phase 1/2	Combined with 5‐fluorouracil‐based regimens/ cetuximab with or without mFOLFOX6/ gemcitabine + nab‐paclitaxel	Revolution Medicines, Inc.	Recruiting	[110b]
RMC‐6236	KRAS G12X	NSCLC, CRC, PDAC	NCT06128551	Phase 1	Combined with RMC‐6291 (KRAS G12C inhibitor)	Revolution Medicines, Inc.	Recruiting	[110]
RMC‐6236	KRAS G12X	NSCLC, CRC, PDAC, Advanced Solid Tumors	NCT05379985	Phase 1	Monotherapy	Revolution Medicines, Inc.	Recruiting	[110b]
YL‐17231	Pan‐KRAS	Advanced Solid Tumors	NCT06096974	Phase 1	Monotherapy	Shanghai YingLi Pharmaceutical Co. Ltd.	Not yet recruiting	[111]
YL‐17231	Pan‐KRAS	Advanced Solid Tumors	NCT06078800	Phase 1	Monotherapy	Shanghai YingLi Pharmaceutical Co. Ltd.	Recruiting	[111]
A synthetic long peptide mutant KRAS vaccine (SPL mKRASvax)	KRAS G12V/A/C/R/D, or G13D	CRC, Pancreatic Cancer	NCT06411691	Phase 2	Combined with Balstilimab and Botensilimab	Sidney Kimmel Comprehensive Cancer Center at Johns Hopkins	Not yet recruiting	[112]
A pooled mutant KRAS peptide vaccine	KRAS G12V/A/C/R/D, or G13D	CRC, PDAC	NCT04117087	Phase 1	Combined With Nivolumab and Ipilimumab	Sidney Kimmel Comprehensive Cancer Center at Johns Hopkins	Recruiting	[113]
A pooled mutant KRAS peptide vaccine	KRAS G12V/A/C/R/D, or G13D	NSCLC	NCT05254184	Phase 1	Combined With Nivolumab and Ipilimumab	Sidney Kimmel Comprehensive Cancer Center at Johns Hopkins	Recruiting	/
A KRAS peptide vaccine	KRAS G12V/A/C/R/D, or G13D	High Risk Cancer, Pancreatic Cancer	NCT05013216	Phase 1	Monotherapy	Sidney Kimmel Comprehensive Cancer Center at Johns Hopkins	Recruiting	[114]
ELI‐002 7P	KRAS G12V/A/C/S/D/R, or G13D	CRC, PDAC	NCT05726864	Phase 1/2	Monotherapy	Elicio Therapeutics	Recruiting	[115]
ELI‐002 2P	KRAS G12R/D	Solid Tumors, Minimal Residual Disease	NCT04853017	Phase 1	Monotherapy	Elicio Therapeutics	Active, not recruiting	[116]
mRNA‐5671/V941	KRAS G12V/C/D, or G13D	NSCLC, CRC, PDAC	NCT03948763	Phase 1	Monotherapy/Combined with Pembrolizumab	Merck Sharp & Dohme LLC	Completed	[117]

^a)^ NSCLC, nonsmall cell lung cancer; CRC, colorectal cancer; PDAC, pancreatic ductal adenocarcinoma.

**Table 2:** Patents of pan‐KRAS inhibitors.

ID	Title	Country	Applicant	Application number	Type
WO2024060966A1	Pan‐KRAS inhibitor compound	CN^a)^	Adlai Nortye Biopharma Co., Ltd.	CN2023116437W	Small molecule inhibitor
WO2024104364A1	Pan‐KRAS inhibitor compound	CN	Adlai Nortye Biopharma Co., Ltd.	CN2023131674W	Small molecule inhibitor
WO2024083256A1	Pan‐KRAS degrading agent, and preparation method therefor and use thereof	CN	Shanghai LeadingTac Pharmaceutical Co., Ltd.	CN2023125974W	Degrader
WO2023169481A1	Tetrahydroisoquinoline derivative as pan‐KRAS inhibitor, preparation method therefor and use thereof	CN	3D Medicines Shanghai Co., Ltd.	CN2023080353W	Small molecule inhibitor
WO2023244599A1	Pan‐KRAS inhibitors	US^b)^	Mirati Therapeutics Inc.	US2023025191W	Small molecule inhibitor
EP4262807A1	Azaquinazoline pan‐KRAS inhibitors	US	Mirati Therapeutics Inc.	EP21907336A	Small molecule inhibitor
WO2024118966A1	Glutarimide‐containing pan‐KRAS‐mutant degrader compounds and uses thereof	US	Tiger Biotherapeutics Inc.	US2023081910W	PPI‐TACs^c)^
EP4262803A1	Tetrahydropyridopyrimidine pan‐KRAS inhibitors	US	Mirati Therapeutics Inc.	EP21907789A	Small molecule inhibitor
WO2024012519A1	Pan‐KRAS inhibitor	CN	Beijing Pharscin Innobio Co., Ltd.	CN2023107172W	Small molecule inhibitor
WO2023150284A2	Quinazoline pan‐KRAS inhibitors	US	Mirati Therapeutics Inc.	US2023012299W	Small molecule inhibitor
CN117720554A	Pan‐KRAS inhibitor compound as well as preparation method and application thereof	CN	Adlai Nortye Biopharma Co., Ltd.	CN202311352357A	Small molecule inhibitor
WO2023137223A1	Pan‐KRAS inhibitors and uses thereof	US; CN	Newave Pharmaceutical Inc.; Guangzhou Lupeng Pharmaceutical Company Ltd	US2023010940W	Small molecule inhibitor
CN117534687A	Pan‐KRAS inhibitor compound	CN	Adlai Nortye Biopharma Co., Ltd.	CN202311516030A	Small molecule inhibitor
WO2023244604A1	Tetrahydropyridopyrimidine pan‐KRAS inhibitors	US	Mirati Therapeutics Inc.	US2023025200W	Small molecule inhibitor
WO2023244615A1	Azaquinazoline pan‐KRAS inhibitors	US	Mirati Therapeutics Inc.	US2023025214W	Small molecule inhibitor
CN117534684A	Pan‐KRAS inhibitor compound	CN	Adlai Nortye Biopharma Co., Ltd.	CN202311516025A	Small molecule inhibitor
CN117534685A	Pan‐KRAS inhibitor compound	CN	Adlai Nortye Biopharma Co., Ltd.	CN202311520023A	Small molecule inhibitor
WO2022258057A1	Compounds as anticancer agents	CN	Jingrui Biopharma Co., Ltd.	CN2022098201W	Small molecule inhibitor
LU505117B1	A pan‐KRAS inhibitor compound	CN	Adlai Nortye Biopharma Co., Ltd.	LU505117A	Small molecule inhibitor
WO2023138589A1	Five‐membered heterocyclic pyrimidine derivative and use thereof as inhibitor of pan‐KRAS mutation	CN	3D Medicines Shanghai Co., Ltd.	CN2023072701W	Small molecule inhibitor
CN116829151A	Azaquinazoline pan KRAS inhibitors	US	Mirati Therapeutics Inc.	CN202180093775A	Small molecule inhibitor
WO2023079320A1	Farnesyl‐transferase inhibitors and KRAS inhibitors for treating KRAS mutant cancers	HU^d)^	Semmelweis Egyetem; Kineto Lab Kft; Eötvös Loránd Tudományegyetem; Természettudományi Kutatóközpont	HU2022050077W	Small molecule inhibitor
CN114933536A	Synthesis method of SOS1 general KRAS inhibitor chiral intermediate	CN	Beijing Lanbote Tech Co., Ltd.	CN202210276336A	Small molecule inhibitor
CN117486901A	Fused piperidine compound, preparation method thereof and application of fused piperidine compound in medicine	CN	Jiangsu Hengrui Medicine Co; Shanghai Hengrui	CN202310957212A	Small molecule inhibitor
CN114874201A (B)	Purple‐KRAS inhibitor as well as preparation and application thereof	CN	3D Medicines Shanghai Co., Ltd.	CN202210370098A	Small molecule inhibitor
JP2023521383A	Methods and compositions using RNA interference and antisense oligonucleotides for inhibiting KRAS	US	University of North Carolina at Chapel Hill	JP2022561542A	RNAi and ASOs^e)^
CN117946135A	Heterocyclic compound, pharmaceutical composition and application thereof	CN	Shanghai Pailong Biotechnology Co., Ltd.	CN202410103584A	Small molecule inhibitor
US2022073923A1	Methods and compositions using RNA interference and antisense oligonucleotides for inhibition of KRAS	US	University of North Carolina at Chapel Hill	US202117532756A	
CN117736226A	Ubiquitous‐KRAS inhibitor and application thereof in medicine	CN	Betta Pharmaceuticals Co., Ltd.	CN202311219388A	Small molecule inhibitor
WO2023099620A1	KRAS degrading compounds comprising annulated 2‐amino‐3‐cyano thiophenes	DE ^f)^; US	Boehringer Ingelheim Int; Univ Vanderbilt	EP2022083950W	Degrader
CN115028644A	SOS1 inhibitor heterocyclic compounds	CN	Shouyao Holdings Beijing Co., Ltd.	CN115028644A	Small molecule inhibitor
CN117466875A	KRAS translation inhibitor as well as preparation method and application thereof	CN	Univ Zhongshan	CN202311407102A	Small molecule inhibitor
CN114853812A	Compound containing phosphine oxide group as well as preparation method and medical application thereof	CN	Sichuan Kelun Botai Biomedical Co., Ltd.	CN202110153855A	Small molecule inhibitor
CN115490699A	Condensed ring compound as well as pharmaceutical composition and application thereof	CN	Chengdu Hyperway Pharmaceutical Co., Ltd.; Shenzhen Haibowei Pharmaceutical Co., Ltd.	CN202210447082A	Small molecule inhibitor
CN117460730A	Pyrido [4, 3‐D] pyrimidine compounds capable of inhibiting KRAS muteins	GB^g)^	Redx Pharma Plc	CN202280038500A	Small molecule inhibitor
JP2024519170A	Compounds	JP^h)^	Redx Pharma Plc	JP2023573109A	Small molecule inhibitor
CN117425658A	Quinazoline derivatives useful as RAS inhibitors	GB	Redx Pharma Plc	CN202280040336A	Small molecule inhibitor
WO2022258974A1	Quinazoline derivatives useful as RAS inhibitors	GB	Redx Pharma Plc	GB2022051446W	Small molecule inhibitor
JP2023525164A	Target‐specific degraders and their medical uses	JP	Oxford University Innovation Ltd	JP2022569162A	Ubiquitin ligase
WO2024115890A1	Compounds	GB	Redx Pharma Plc	GB2023053075W	Small molecule inhibitor
WO2023034537A1	Compositions and methods for modulating KRAS expression	US	Molecular Axiom LLC	US2022042393W	A composition comprising antisense oligonucleotides capable of binding to KRAS mRNA.
CN118251494A	Compositions and methods for modulating KRAS expression	US	Molecular Axiom	CN202280072704A	A composition comprising antisense oligonucleotides capable of binding to KRAS mRNA.
WO2024034591A1	Heterocyclic compound for inhibiting and/or inducing degradation of KRAS protein	JP	Astellas Pharma Inc.	JP2023028860W	Small molecule inhibitor
WO2024035921A1	Degraders of son of sevenless homolog 1	US	H. Lee Moffitt Cancer Center & Research Institute;Yuan Yu	US2023030058W	Degrader
KR20230087570A	Pan‐RAS mRNA cancer vaccine	US	RNAimmune Inc.	KR20237016255A	mRNA vaccine
WO2024030633A1	Compositions and methods for inhibition of KRAS	US	Theras Inc.; Leidos Biomedical Research Inc.; Lawrence Livermore National Security LLC	US2023029520W	Small molecule inhibitor
JP2024522215A	Multiplexed TP53 and pan‐RAS mRNA cancer vaccine	US	RNAimmune Inc.	JP2023577149A	mRNA vaccine
WO2024029613A1	Heterocyclic compound for inducing degradation of mutant KRAS protein	JP	Astellas Pharma Inc.	JP2023028510W	Small molecule inhibitor
WO2024104453A1	Fused tricyclic compound, preparation method therefor, and pharmaceutical use thereof	CN	Jiangsu Hengrui Pharmaceuticals Co., Ltd.; Shanghai Hengrui Pharmaceuticals Co., Ltd.	CN2023132224W	Small molecule inhibitor
WO2024040131A1	Pyridopyrimidine KRAS inhibitors	US	Treeline Biosciences Inc.	US2023072332W	Small molecule inhibitor
WO2024112654A1	Spirocyclic dihydropyranopyrimidine KRAS Inhibitors	US	Treeline Biosciences Inc.	US2023080513W	Small molecule inhibitor

^a)^ CN, China;

^b)^ US, United States;

^c)^ PPI‐TAC, protein‐protein interaction targeted chimeras;

^d)^ HU, Hungary;

^e)^ RNAi, RNA interference; ASOs, antisense oligonucleotides;

^f)^ DE, Deutschland;

^g)^ GB, Great Britain;

^h)^ JP, Japan.

The understanding of the TME related with KRAS mutations and treatment represents another obstacle in the multi‐KRAS era. Emerging evidence showed that differences and similarities existed among the TME of tumors with wild‐type and oncogenic KRAS mutants. Comprehending these characteristics will be helpful to the treatment of KRAS‐driven tumors. However, no existing review articles have summarized the immune, metabolic, stromal, and microbial microenvironments related with KRAS mutations and their inhibitors. The following paragraphs focused on combining the existed evidence about the KRAS‐related TME and analyzing potential TME targets for combination therapy.

## 3 The Impact of KRAS Mutations and Their Inhibitors on the Immune Microenvironment

**Figure 3:**
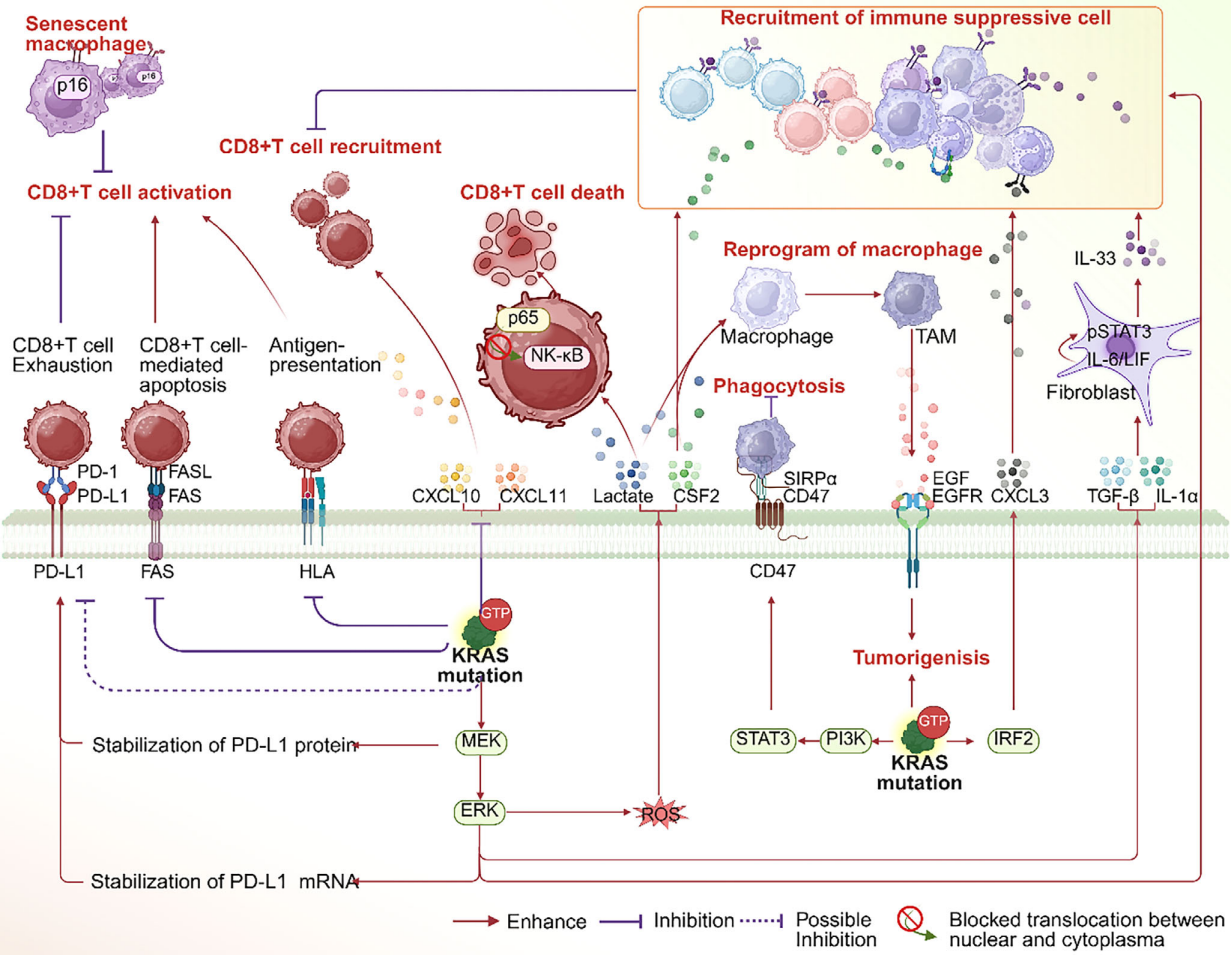
Alterations in the tumor immune microenvironment induced by oncogenic KRAS mutations.

The tumor immune microenvironment consists of immune cells infiltrating the tumor stroma as well as the cytokines they secrete. Tumor cells, in conjunction with their stroma, shape an immune microenvironment that promotes tumor development by modulating immune cell infiltration and altering the functional capacity of infiltrating immune cells. A growing body of research has demonstrated that tumors with common mutations, such as KRAS G12C and KRAS G12D, typically exhibit an immune microenvironment characterized by reduced CD8+ T cell presence and functional exhaustion, coupled with an increase in immunosuppressive cells (**Figure** **3**).

This figure presented the implications of oncogenic KRAS mutations on the recruitment, activation, reprogramming, or death of immune cells. It emphasized the roles of CD8+ T cells and immune suppressive cells. Key molecules mediating these processes were also highlighted.

KRAS‐mutant tumors demonstrated markedly diminished CD8+ T cell infiltration compared to their wild‐type counterparts. Importantly, elevated mutant KRAS expression showed an inverse correlation with intratumoral CD8+ T cell density.^[^ ^38^ ^]^ Stratification of KRAS mutation subtypes in CRC showed no significant differences in the intratumoral CD8+ T cell density across different KRAS mutation types (e.g., KRAS G12D, G12V, G13D, and others).^[^ ^38b^ ^]^ In PDAC, variations in the spatial distribution of CD8+ T cells may arise due to specific KRAS mutations such as G12D and G12R. For instance, KRAS G12D tumors exhibited more niches rich in T cells but largely devoid of tumor cells, a characteristic associated with poorer clinical outcomes. In contrast, KRAS G12R tumors contained more niches where T cells and tumor cells coexisted, correlating with better clinical prognosis.^[^ ^39^ ^]^ This observation suggested that KRAS G12D may hinder antitumor immunity by restricting effective interactions between CD8+ T cells and tumor cells. Further research is necessary to substantiate this hypothesis.

After activation, CD8+ T cells differentiate into effector T cells, which are capable of specifically recognizing and binding to tumor cells. These effector T cells eliminate tumor cells by releasing cytolytic substances, such as perforin and granzyme, or by triggering apoptosis through the FAS‐FASL death signaling pathway. In vivo experiments demonstrated that KRAS mutations contributed to the reduced intratumoral density of effector T cells.^[^ ^38b^ ^]^ Mechanistically, KRAS mutations impeded the generation of intratumoral effector T cells by suppressing the chemotaxis and activation of CD8+ T cells. For instance, KRAS G12D suppressed the levels of cancer cell‐derived CXCL10 and CXCL11, thus hindering CD8+ T cell infiltration.^[^ ^40^ ^]^ Additionally, KRAS G12D upregulated IL‐33 expression in tumor cells via the RAF/MEK/ERK signaling pathway.^[^ ^41^ ^]^ It also enhanced the secretion of TNF‐α and IL‐1α by tumor cells, initiating the autocrine signaling pathway to activate pSTAT3 in fibroblasts.^[^ ^42^ ^]^ The active pSTAT3 signals subsequently induced IL‐33 expression in fibroblasts.^[^ ^42^ ^]^ IL‐33, through its receptor ST2, modulated the secretion profiles of innate lymphoid cells, regulatory T cells (Tregs), and myeloid cells, thereby inhibiting CD8+ T cell infiltration and activation.^[^ ^42^ ^]^ Several factors contribute to the modulation of CD8+ T cell activity. Beyond the immunosuppressive cytokines secreted by the aforementioned cells, other crucial inhibitory mechanisms included impaired antigen processing and presentation, suppression of the FAS‐FASL signaling pathway, and enhancement of the PD‐1‐PD‐L1 axis. KRAS G12V signaling significantly downregulated molecules involved in antigen processing and presentation.^[^ ^43^ ^]^ Moreover, KRAS mutations epigenetically silenced FAS gene. As a result, the ability of CD8+ T cells to induce apoptosis in tumor cells via the FAS‐FASL pathway was markedly diminished.^[^ ^44^ ^]^ The activation of the oncogenic RAS/MEK/ERK signaling pathway, driven by various KRAS mutations, stabilized both the mRNA and protein of PD‐L1.^[^ ^43^, ^45^ ^]^ KRAS G12V was reported to upregulate PD‐L1 expression via the TGF‐β/ epithelial‐mesenchymal transition (EMT) signaling pathway.^[^ ^46^ ^]^ However, the regulation of PD‐L1 expression was complex, and its association with KRAS mutations was not always positive. Conflicting findings have been reported, particularly in KRAS G12D‐driven NSCLC.^[^ ^47^ ^]^ In this subtype, PD‐L1 expression was lower in both tumor cells and immune cells compared to non‐G12D‐driven KRAS NSCLC. This discrepancy may be attributed to distinct comutations that occur at relatively high frequencies in NSCLC.^[^ ^48^ ^]^

Additionally, KRAS mutations facilitated the depletion of intratumoral effector T cells by inducing their apoptosis. They enhanced lactate production, resulting in the translocation of circATXN7 from the nucleus to the cytoplasm in cytotoxic CD8+ T cells. In the cytoplasm, circATXN7 bond to the p65 subunit of NF‐κB, effectively sequestering it and thereby promoting the death of cytotoxic CD8+ T cells. Notably, this mechanism appeared to operate independently of the specific KRAS mutation subtype.^[^ ^38^, ^49^ ^]^

Tregs, tumor‐associated macrophages (TAMs), and myeloid derived suppressive cells (MDSCs) were the primary immune suppressor cell populations enriched in KRAS mutant tumors. Clinical specimens showed that CRC with KRAS mutations exhibited a higher density of Tregs compared to wild‐type tumors.^[^ ^50^ ^]^ In vivo experiments demonstrated that KRAS G12C, G12D, and G12V promoted the secretion of IL‐10 and TGF‐β1 through the MEK‐ERK signaling pathway, facilitating the conversion of CD4+ T cells into Treg phenotypes.^[^ ^50^, ^51^ ^]^ Macrophages, key components of the tumor immune microenvironment, can switch between pro‐tumor and antitumor states. Mutations such as KRAS G12C, G12D, and G12V drove the reprogramming of macrophages into protumor TAMs, with a significantly greater effect compared to wild‐type KRAS.^[^ ^52^ ^]^ Mechanistically, oncogenic KRAS signaling enabled immune evasion in lung adenocarcinoma by activating CD47.^[^ ^53^ ^]^ KRAS mutations increased reactive oxygen species (ROS) production, which stabilizes HIF‐1α, thereby promoting the production of CSF2 and lactate by tumor cells. This combination of CSF2 and lactate reprogramed macrophages into a TAM‐like phenotype.^[^ ^52a^ ^]^ KRAS G12D PDAC cells also transferred mutant KRAS protein to macrophages via exosomes, promoting their TAM phenotype through a STAT3‐dependent pathway.^[^ ^54^ ^]^ Oncogenic KRAS activated PI3K/STAT3 to inhibit miR‐34a expression, thereby reducing the post‐transcriptional suppression of CD47 by miR‐34a. Upregulated CD47 in cancer cells suppressed macrophage phagocytic ability.^[^ ^53^ ^]^ TAMs further promoted the tumorigenic transformation of KRAS G12D‐driven pancreatic intraepithelial neoplasia (PanIN) via the EGF/EGFR signaling pathway.^[^ ^52^, ^55^ ^]^ Macrophages also regulated cytotoxic T cell‐mediated antitumor immunity. For example, in lung tissues from KRAS G12D mutant mice and early‐stage NSCLC adenocarcinoma patients, senescent alveolar macrophages with high p16 and CXCR1 expression accumulated. In vivo, clearing these cells enhanced antitumor responses by promoting the accumulation of cytotoxic T cells, delaying tumor initiation and progression.^[^ ^56^ ^]^ MDSCs are known for their immunosuppressive function.^[^ ^57^ ^]^ In CRC and PDAC, KRAS mutations promoted MDSC infiltration, contributing to an immunosuppressive tumor microenvironment.^[^ ^44^, ^58^ ^]^ Mechanistically, KRAS G12D inhibited IRF2, promoting CXCL3 expression, which bond to CXCR2, facilitating MDSC accumulation.^[^ ^58^ ^]^ In KRAS G12V lung cell lines, an upregulation of myeloid regulatory factors like IL‐8, CXCL1, CCL2, and CSF2, along with the suppression of the interferon pathway, was observed. This suggested oncogenic KRAS signaling could enhance myeloid chemokines and growth factors, promoting the infiltration of myeloid suppressor cells.^[^ ^59^ ^]^

KRAS inhibitors enhanced interferon signaling and promoted antigen presentation signals by inhibiting oncogenic KRAS signaling. This led to increased infiltration and activity of CD8+ T cells and antigen‐presenting cells, while simultaneously suppressing the infiltration and function of immune suppressor cells.^[^ ^11^, ^12^, ^38^, ^44^, ^59^, ^60^ ^]^ Notably, they did not significantly affect CD8+ T cell infiltration in KRAS wild‐type tumors.^[^ ^38b^ ^]^ For example, the multi‐RAS inhibitor RMC‐7977 promoted the infiltration of tumor antigen‐specific CD8+ T cells.^[^ ^12a^ ^]^ And the pan‐KRAS degrader TKD induced an increase in IRF2 and a decrease in PD‐L1 in tumor cells, significantly increasing the density of effector T cells and reducing the density of MDSCs in KRAS G12D mutant CRC tumors in mice. This results in significant suppression of tumor growth and metastasis, as well as enhanced sensitivity to immune checkpoint therapy and EGFR antibodies.^[^ ^11c^ ^]^ In another study, conditional elimination of KRAS G12D or the use of a KRAS G12D inhibitor led to an enhancement of major histocompatibility complex (MHC) II antigen presentation signaling, resulting in increased infiltration and activation of antigen‐presenting cells, CD4+ T cells, and CD8+ T cells. This enhancement also augmented the FAS‐FASL‐mediated cytotoxic effects of CD8+ T cells on tumors, suggesting an enhancement of adaptive antitumor immunity. The KRAS G12C inhibitor AMG 510 downregulated CD47 expression by disrupting the KRAS/miR‐34a/CD47 signaling axis, thereby reducing the ability of immunosuppressive checkpoints to inhibit the phagocytic capacity of macrophages.^[^ ^53^ ^]^ Furthermore, conditional elimination of KRAS G12D resulted in a reduction of exhausted TIM3+ CD4+ T cell and MDSC frequencies, a decrease in PD‐L1 expression within myeloid cells, and a reduction in FAS‐mediated apoptosis of CD8+ T cells, indicating a weakening of immunosuppressive signals.^[^ ^44^, ^60^ ^]^ KRAS G12C inhibitors further enhanced interferon signaling by suppressing MYC, leading to an increase in MHC I expression on tumor cells, enhanced infiltration and activation of antigen‐presenting dendritic cells and CD8+ T cells, and a reduction in the infiltration of immunosuppressive cells, thereby providing a strong foundation for adaptive antitumor immunity.^[^ ^59^ ^]^ At the same time, KRAS G12C inhibitors reduced the infiltration of MDSCs and increased the infiltration of proinflammatory macrophages, thereby playing a beneficial regulatory role in antitumor immunity.^[^ ^60^, ^61^ ^]^ Mechanistically, KRAS G12C inhibitors relieved the suppression of the interferon signal by MYC, enhancing the expression of interferon‐driven immune regulatory genes.^[^ ^59^ ^]^ This led to an increase in the expression of T cell chemokines, such as CXCL9, CXCL10, CXCL11, and CXCL13, while suppressing the levels of MDSC chemokines, such as CCL2, CXCL1, CXCL2, CXCL3, and CCL9.^[^ ^60^, ^61^ ^]^

## 4 The Impact of KRAS Mutations and Their Inhibitors on the Metabolic and Stromal Microenvironment

**Figure 4:**
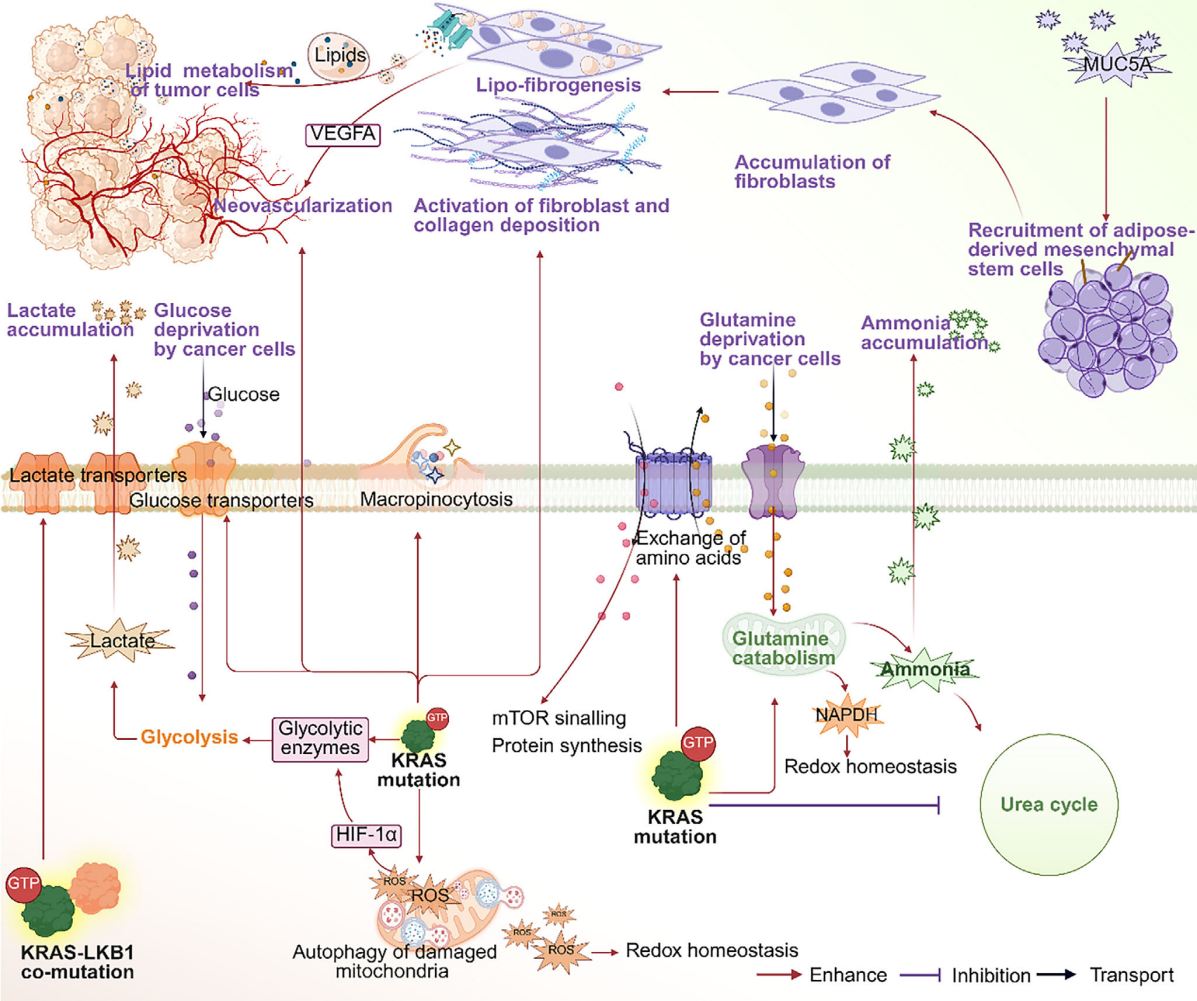
Alterations in the tumor metabolic and stromal microenvironment induced by oncogenic KRAS mutations.

Oncogenic KRAS signaling exerts extensive influence on glucose, lipid, and amino acid metabolism, as well as macropinocytosis in tumor cells. Mutant KRAS enhanced glycolysis and lactate production by upregulating the expression and activity of glucose transporters^[^ ^62^ ^]^ and key glycolytic enzymes.^[^ ^63^ ^]^ Additionally, KRAS mutations mitigated excessive ROS levels by inducing autophagy of mitochondria to remove defective mitochondria.^[^ ^64^ ^]^ Furthermore, these mutations boosted macropinocytosis, which was an endocytic process nonselectively internalized extracellular fluid, nutrients, or pathogens. It facilitated the uptake of extracellular nutrients,^[^ ^65^ ^]^ and promote glutamine catabolism,^[^ ^66^ ^]^ and lipid biosynthesis^[^ ^67^ ^]^ in tumor cells. The impacts and mechanisms of mutant KRAS on these metabolic processes have been comprehensively reviewed in previous studies.^[^ ^15^, ^68^ ^]^ This section aims to summarize and analyze the evidence on how KRAS mutations and their inhibitors influence the metabolic and stromal microenvironments (**Figure** **4**).

This figure illustrated the impact of oncogenic KRAS mutations on the tumor metabolic and stromal microenvironment and its related tumor cell metabolism. Key metabolic characteristics of KRAS mutant tumors included the accumulation of lactate and ammonia, and the depletion of glutamine and glucose. Additionally, the accumulation and activation of fibroblasts, along with neovascularization, were stromal features associated with KRAS mutations.

KRAS mutations result in the accumulation of lactate and ammonia, and depletion of glutamine in the tumor microenvironment. Oncogenic KRAS elevated ROS, which stabilized hypoxia‐inducible factors. These factors promoted glycolysis in tumor cells, leading to enhanced lactate synthesis and secretion.^[^ ^49^, ^52^ ^]^ In lung adenocarcinoma, KRAS mutations frequently cooccur with loss‐of‐function mutations in the LKB1 gene. The comutation of KRAS and LKB1 elevated the expression of the lactate transporters in tumor cells. And these transporters resulted in increased lactate export.^[^ ^69^ ^]^ Glutamine is an essential amino acid that acts as a donor of nitrogen and carbon. It supports rapid proliferation and survival of tumor cells. KRAS mutations enhanced glutamine uptake and metabolism in tumor cells through various mechanisms. It helped tumor cells to exacerbate glutamine deficiency in the microenvironment.^[^ ^66^, ^70^ ^]^ Mechanistically, KRAS mutations upregulated the expression of glutaminase, which promoted enzymatic conversion of glutamine to downstream products.^[^ ^71^ ^]^ Additionally, KRAS mutations increased the expression of aspartate transporters to facilitate the transport of mitochondrial glutamine‐derived aspartate into the cytosol. This process provided metabolic precursors for NADPH, which played an important role in cellular redox homeostasis and macromolecule synthesis.^[^ ^66c^ ^]^ KRAS G12D also upregulated glutamine transporters. In the intestinal epithelium with KRAS G12D mutations, glutamine was exported via glutamine transporters in exchange for essential amino acids, thereby sustaining mTOR signaling and protein synthesis.^[^ ^72^ ^]^ Ammonia is produced from glutamine catabolism. Ammonia enters the urea cycle to synthesize arginine and urea. Dysregulation of the urea cycle was a significant metabolic alteration that promoted cancer.^[^ ^73^ ^]^ In CRC, KRAS G12D mutations downregulated the expression of HNF4α and OTC. HNF4α was a key regulatory factor essential for ammonia clearance in the urea cycle, while OTC catalyzed a critical step in the conversion of ammonia to urea. Deficiencies in both HNF4α and OTC led to ammonia accumulation in the TME.^[^ ^74^ ^]^

The tumor stromal microenvironment comprises primarily adipocytes, fibroblasts, vascular endothelial cells, and collagen fibers, along with their cellular products. In pancreatic intraepithelial neoplasia, KRAS mutations played a dominant role in shaping the stromal component. KRAS G12D activated pancreatic stellate cells by upregulating SHH secretion, which was crucial for forming the stroma‐rich microenvironment.^[^ ^75^ ^]^ In KRAS G12D PDAC mice, the tumor‐secreted protein MUC5A was significantly enriched in the TME. It promoted the recruitment of adipose‐derived mesenchymal stem cells. These cells then facilitated the accumulation of fibroblasts.^[^ ^76^ ^]^ In a CRC mouse model driven by oncogenic KRAS mutations, KRAS activation stimulated the transcription of proadipogenic factors. This induced the conversion of fibroblasts into a lipid‐rich phenotype. These lipid‐rich fibroblasts produced vascular endothelial growth factor A (VEGFA), promoting angiogenesis and tumor progression.^[^ ^77^ ^]^ Lipid‐rich fibroblasts were also observed in KRAS‐driven PDAC. These lipid‐rich fibroblasts promoted the efflux of lipids, cholesterol, and taurocholic acid into the microenvironment. Tumor cells took these lipids to support tumor growth.^[^ ^78^ ^]^ TGF‐β, which could be up‐regulated by KRAS mutations, enhanced the transcription of fibrogenic factors in fibroblasts, and therefore contributed to collagen deposition.^[^ ^79^ ^]^ Grunwald et al.^[^ ^80^ ^]^ characterized the subtumor microenvironment (subTME) of pancreatic cancer, suggesting that certain pancreatic cancer patients exhibited intratumoral heterogeneity in their microenvironments. They demonstrated that this heterogeneity was closely associated with fibroblasts. Although there was no evidence suggesting that KRAS shaped this subTME, considering its role in modulating fibroblasts phenotypes, it is worthwhile exploring the potential regulatory effects of KRAS mutations on the subTME described in the study. In lung cancer, KRAS mutations could regulate the transcription of angiogenesis‐related genes, ultimately leading to tumor neovascularization.^[^ ^81^ ^]^ These regulatory effects of KRAS mutations on the stromal microenvironment provide new mechanistic insights into the protumorigenic roles of KRAS.

Evidence regarding the effects of KRAS inhibitors on the metabolic and stromal microenvironment is limited. Bian and colleagues demonstrated that KRAS G12C inhibitors significantly upregulated the gene expression and enzymatic activity of aldehyde dehydrogenase 1 family member A1 (ALDH1A1).^[^ ^82^ ^]^ The enzymatic activity of ALDH1A1 was key for mediating resistance to KRAS G12C inhibitors. Knockout of ALDH1A1 enhanced the sensitivity of KRAS G12C tumors to both KRAS G12C and pan‐KRAS inhibitors by inducing ferroptosis in tumor cells. It also increased the sensitivity of KRAS G12D mutant tumors to KRAS G12D inhibitors. Han and colleagues discovered that in KRAS mutant pancreatic tumors, fibroblast‐derived NRG1 activated the ERBB2/ERBB3 pathway in tumor cells, enabling them to grow independently of KRAS signaling and leading to resistance against KRAS inhibitors.^[^ ^83^ ^]^ Following KRAS inhibitor treatment, the expression of ERBB2/3 was significantly increased highlighting the unique role of fibroblasts in conferring resistance to KRAS inhibitors. These two studies underscore the necessity and value of investigating the metabolic and stromal changes associated with KRAS inhibitors. Future research should further explore the alterations in the metabolic and stromal microenvironment induced by KRAS inhibitors.

## 5 The Impact of KRAS Mutations and Their Inhibitors on the Microbe Microenvironment

**Figure 5:**
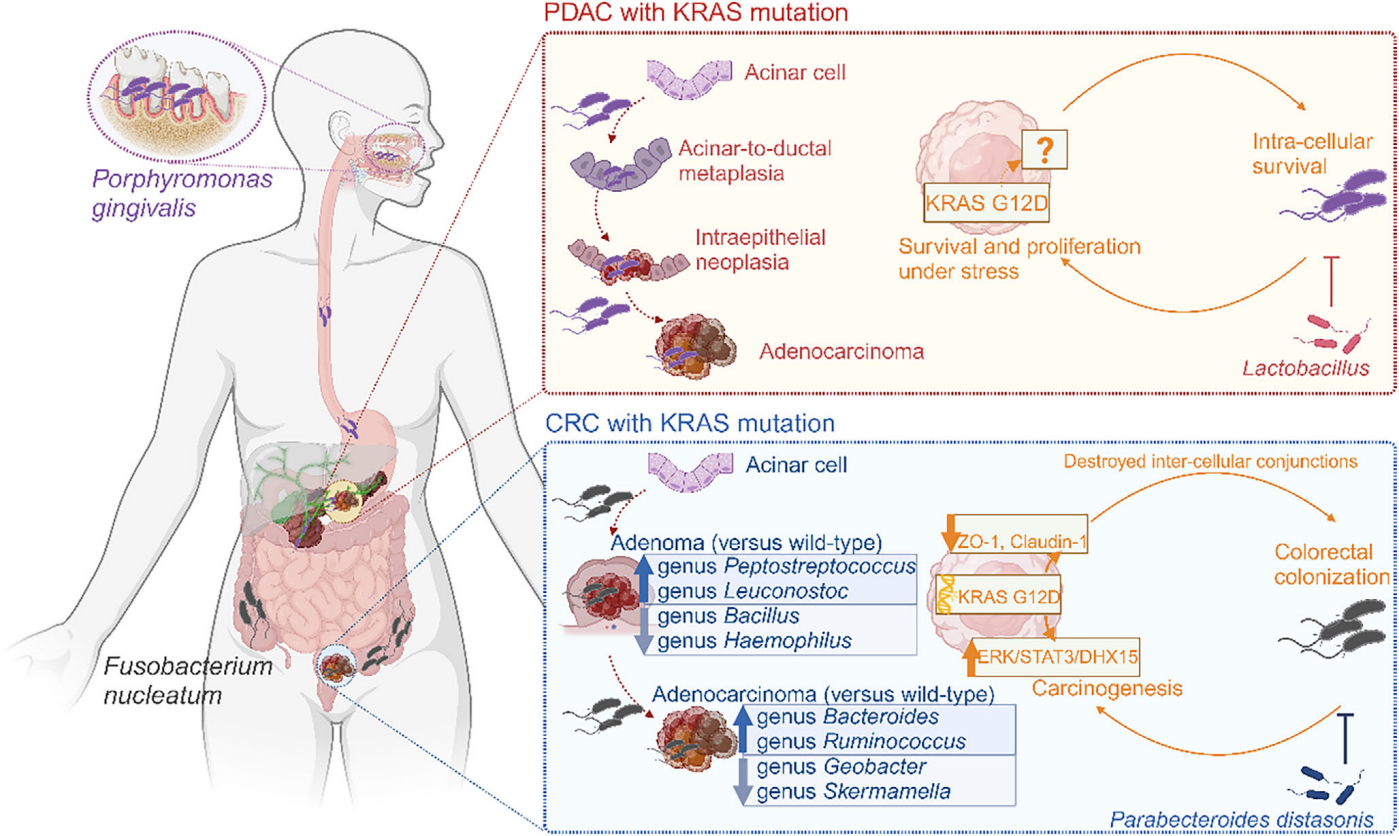
The tumor microbial microenvironment associated with oncogenic KRAS mutations.

The presence of intratumoral microbes and their impact on tumor evolution have gained attention, offering new avenues for cancer treatment.^[^ ^84^ ^]^ Cancers with prevalent KRAS mutations, such as those in the digestive tract, lungs, and melanomas, tended to show enriched microbial communities.^[^ ^2^, ^84^ ^]^ Compared to their wild‐type counterparts, KRAS‐mutated tumors exhibited distinct microbial landscapes. Evidence of these unique microbial environments has primarily emerged from studies on colorectal and pancreatic cancers. These cancers shared notable characteristics: they could take years or even decades and go through several pathological stages from precancerous lesions to invasive tumors,^[^ ^85^ ^]^ exhibited prevalent KRAS mutations,^[^ ^2^ ^]^ contacted with microbes through natural cavities.^[^ ^86^ ^]^ Given the similar distributions and influential roles of microbes and KRAS mutations in tumor,^[^ ^7^, ^86^, ^87^ ^]^ it is necessary to delineate the microbial landscape within KRAS‐mutated tumors and reflect on their crosstalk, so as to provide clues for microbe‐based precision medicine (**Figure** **5**).

Evidence connecting the tumor microbial microenvironment with oncogenic KRAS mutations was limited. Microbial components varied significantly by cancer type; therefore, this figure focuses on pancreatic adenocarcinoma (PDAC) and colorectal cancer (CRC) separately.

CRC typically follows the adenoma‐adenocarcinoma sequence. In adenomas, KRAS‐mutated types showed a higher enrichment of genus *Peptostreptococcus* and *Leuconostoc* and a lower abundance of genus *Bacillus* and *Haemophilus* compared to wild‐type adenomas. The classification model built using these KRAS mutation‐associated genera achieved an area under the curve (AUC) of 0.83. For adenocarcinomas, KRAS mutations led to a higher enrichment of genus *Bacteroides, Ruminococcus, and Peptostreptococcus*, while genus *Geobacter and Skermanella* were less enriched compared to their wild‐type counterparts. The classification model for KRAS in adenocarcinomas, based on these microbial associations, achieved an AUC of 0.90.^[^ ^88^ ^]^ This study provided a good example for the research of KRAS‐associated microbiota. First, it conducted biopsies from multiple sites, effectively illustrating the average distribution of genotypes and microorganisms within tumors, thereby minimizing the impact of intratumoral heterogeneity on the results. Second, it explored the changes in microbial composition from adenoma to adenocarcinoma and described the correlation between KRAS mutations and microorganisms. However, the sample size in this study was small, which hindered the ability to reveal the differences of microbial compositions among KRAS variants. In a clinical cohort of 263 pairs of CRC tumors and adjacent noncancerous tissues, researchers found a significantly higher abundance of *Fusobacterium nucleatum* in KRAS‐mutated tissues compared to both adjacent tissues and KRAS wild‐type tissues. When categorizing by KRAS mutation variants, they observed that tumors with KRAS G12D (*n* = 5) exhibited a significantly higher *Fusobacterium nucleatum* abundance than those with wild‐type KRAS (*n* = 12), while tumors with KRAS G13D (*n* = 3) and other variants (*n* = 3) did not show statistical differences, likely due to the small sample size.^[^ ^89^ ^]^ In animal models, exogenous *Fusobacterium nucleatum* preferentially accumulated in the colonic tissues of KRAS G12D mutant mice rather than in wild‐type mice. The tumor‐promoting effects of *Fusobacterium nucleatum* were significantly stronger in KRAS G12D mutant mice compared to wild‐type mice. In vitro studies with tumor cells and organoids revealed that *Fusobacterium nucleatum* exhibited a higher invasive ability in KRAS G12D mutant tumor cells than in KRAS G13D or wild‐type cells. Inhibiting KRAS G12D reduced the invasiveness. Mechanistically, KRAS G12D decreased the expression of tight junction proteins ZO‐1 and Claudin‐1, compromising the intestinal epithelial barrier and facilitating *Fusobacterium nucleatum* invasion. Additionally, KRAS G12D activated ERK‐STAT3 signaling, upregulating nuclear DHX15 expression. DHX15 bound to FN1859 on the surface of *Fusobacterium nucleatum*, mediating the tumor‐promoting role of *Fusobacterium nucleatum* in KRAS G12D mutant tumors.^[^ ^89^ ^]^

PDAC typically follows the acinar‐to‐ductal metaplasia (ADM) – PanIN – PDAC sequence.^[^ ^90^ ^]^ KRAS mutations and microbes work together to drive this process. One well‐studied microorganism is *Porphyromonas gingivalis*. Commonly found in the subgingival plaque of patients with periodontal disease, *Porphyromonas gingivalis* migrated from the oral cavity to the pancreas. Prolonged exposure to this bacterium induced ADM in healthy wild‐type mice.^[^ ^87a^ ^]^ The KRAS G12D mutation is a pivotal factor driving the transition from ADM to PanIN, and finally to PDAC.^[^ ^7^, ^91^ ^]^ In KRAS G12D mutant mice, *Porphyromonas gingivalis* further accelerated the progression from PanIN to PDAC.^[^ ^87^, ^92^ ^]^ Mechanistically, *Porphyromonas gingivalis* invaded acinar and PDAC cells, promoting cellular proliferation.^[^ ^87^, ^93^ ^]^ The KRAS G12D enhanced the intracellular survival of *Porphyromonas gingivalis*, while the bacterium, in turn, promoted the survival and proliferation of KRAS G12D tumor cells under conditions of glucose deprivation and oxidative stress. Together, they formed a beneficial relationship that accelerated the onset of PDAC.^[^ ^87a^ ^]^

The microbial microenvironment of KRAS mutant tumors is a complex network where microorganisms influence each other. Chronic *Porphyromonas gingivalis* infection not only promoted the progression of pancreatic cancer in KRAS G12D mice, but also led to changes in the pancreatic microbiome, which was manifested by the increase of *Mycoplasma pancrea*, *Helibacterium* and *Paraprevobacteriaceae*.^[^ ^87a^ ^]^ Administration of *Lactobacillus* partially alleviated the procancer effect of *Porphyromonas gingivalis*.^[^ ^92^ ^]^ In CRC mice with the KRAS G12D mutation, the enrichment of *Fusobacterium nucleatum* led to the depletion of *Parabacteroides distasonis*. Conversely, supplementing with *Parabacteroides distasonis* curtailed the invasiveness of *Fusobacterium nucleatum* and decelerated the tumor progression driven by it. Clinically, *Parabacteroides distasonis* and *Fusobacterium nucleatum* were negatively correlated in KRAS G12D samples, a relationship not observed in wild‐type ones, indicating that the competitive interactions were specific to KRAS G12D mutations. However, the underlining mechanisms require further elucidation.^[^ ^89^ ^]^

Research on intratumoral microbiomes and the development of KRAS inhibitors did not gain significant momentum until 2021. Consequently, there are currently no studies reporting the impact of KRAS inhibitors on the tumor microbial microenvironment. Given that cancer types with prevalent KRAS mutations exhibited a diverse microbial composition, and that intratumoral microbiota could contribute to resistance against chemotherapy and immunotherapy,^[^ ^94^ ^]^ it is essential for future studies to explore changes in microbial composition associated with KRAS inhibitor treatment, and to identify specific intratumoral microbial species linked to treatment response or resistance. Such investigations could offer valuable insights for the treatment of KRAS mutant tumors.

## 6 The Crosstalk Network Among Different Kinds of Microenvironment

**Figure 6:**
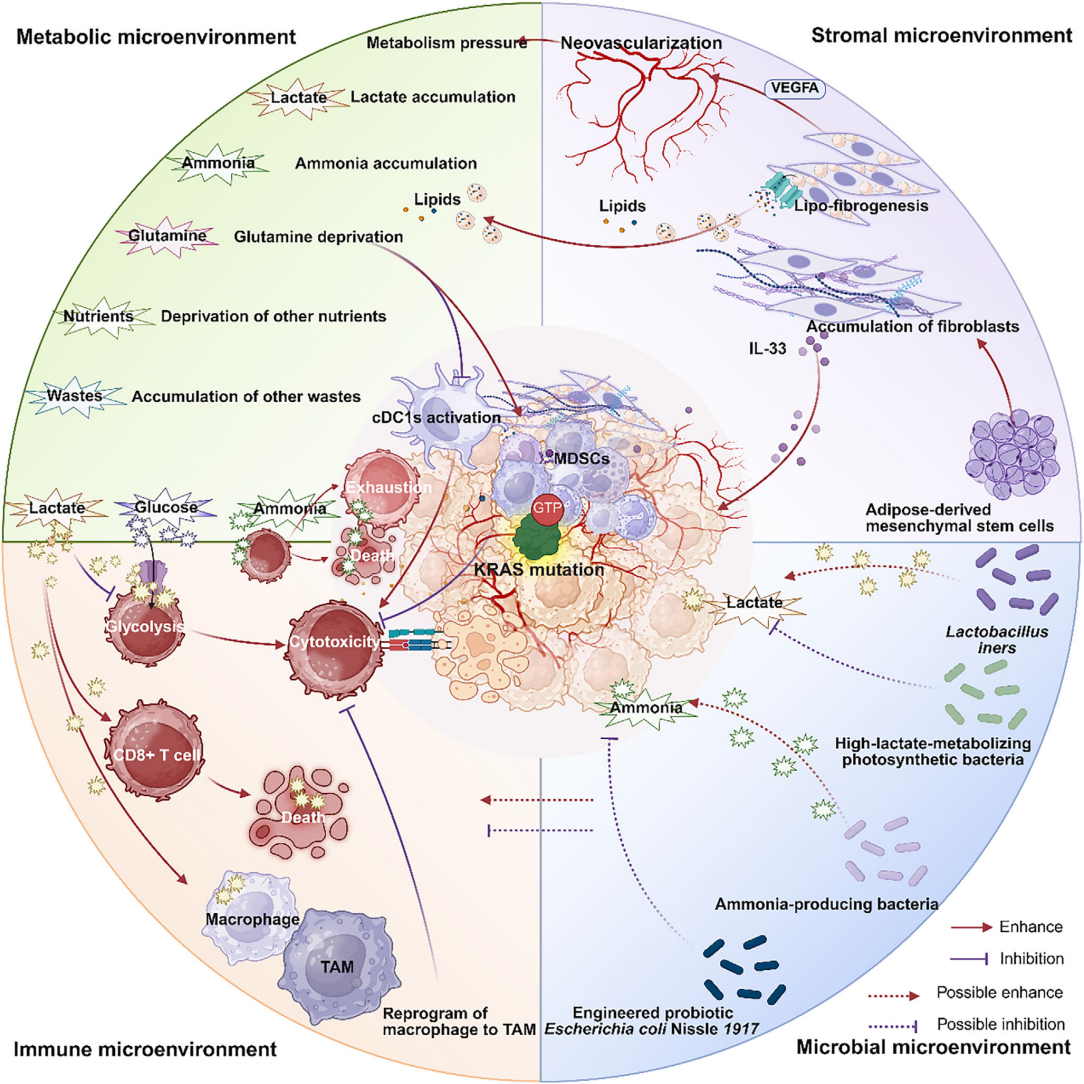
The crosstalk among tumor microenvironment residents in KRAS mutant tumors.

The various components within the TME engage in dynamic reciprocal crosstalk (**Figure** **6**). The metabolic stresses induced by KRAS mutations, which have been discussed in Section 5, could reshape the phenotypes and functions of immune cells. For example, excessive lactate drove macrophage polarization towards the M2 type, which in turn reduced T cell infiltration and suppressed T cell function.^[^ ^52^, ^69^ ^]^ Lactate weakened the antitumor effects of CD8+ T cells by binding to their glucose transporters and inhibiting glycolysis.^[^ ^95^ ^]^ Moreover, lactate could enhance the activation‐induced cell death in CD8+ T cells by suppressing nuclear translocation of NF‐κB.^[^ ^49^ ^]^ Elevated ammonia refluxed into mitochondria, leading to mitochondrial damage and T cell death.^[^ ^96^ ^]^ Additionally, ammonia induced oxidative stress and exhaustion in T cells by inducing their metabolic reprogramming.^[^ ^74^ ^]^ The deficiency of glutamine impaired the maturation and activation of cDC1s and promoted the immunosuppressive phenotype of myeloid cells, which ultimately compromised the antitumor function of CD8+ T cells.^[^ ^70^ ^]^

This figure presented the crosstalk among different microenvironment residents in KRAS mutant tumors, based on current evidence. Most of the present research highlighted the interactions between the immune and metabolic microenvironments. Communications among other components remained largely unexplored and warrants further investigation.

Stromal cells, such as fibroblasts, can also regulate microenvironment phenotypes. For example, fibroblasts secreted IL‐33 to promote the infiltration of immunosuppressive cells and alter their secretory phenotype. This further suppressed CD8+ T cell chemotaxis.^[^ ^42^ ^]^ Lipidized fibroblasts released lipids and VEGFA to promote neovascularization and tumor lipid metabolism.^[^ ^77^ ^]^ Tumor neovascularization refers to the formation of abnormal blood vessels that may further exacerbate metabolic stress and immune suppression within the microenvironment.^[^ ^97^ ^]^

The bacterial components in the tumor microenvironment can influence the metabolic and immune microenvironments.^[^ ^14d^ ^]^ For example, *Lactobacillus iners* were enriched in cervical cancer tumor tissues and produced lactate through anaerobic glycolysis.^[^ ^98^ ^]^ A high‐lactate‐metabolizing photosynthetic bacteria (LAB‐1) selectively colonized the TME and consumed lactate, significantly reducing lactate levels in tumor tissues. This alleviated tumor‐induced immunosuppression and promoted the infiltration and activation of various antitumor immune cells.^[^ ^99^ ^]^ Ammonia can also be produced or cleared by bacteria. Researchers developed an engineered probiotic strain of *Escherichia coli* Nissle 1917 using synthetic biology methods, which integrated the exogenous N‐acetylglutamate synthase (ArgA) gene to enhance the urea cycle, resulting in ammonia clearance. This enhancement increased the number of tumor‐infiltrating T cells and exhibited a significant synergistic effect with PD‐L1 blocking antibodies in tumor clearance.^[^ ^100^ ^]^ The impact of tumor‐colonizing bacteria on the tumor immune microenvironment had been extensively summarized in published reviews.^[^ ^14d^ ^]^ However, there is currently limited evidence directly linking microbiome‐metabolism‐immune interactions to KRAS mutations, highlighting the need for further research in this area.

## 7 Targeting Strategies for the Microenvironment and Their Combinations with KRAS Inhibitors: From Bench to Bed

**Figure 7:**
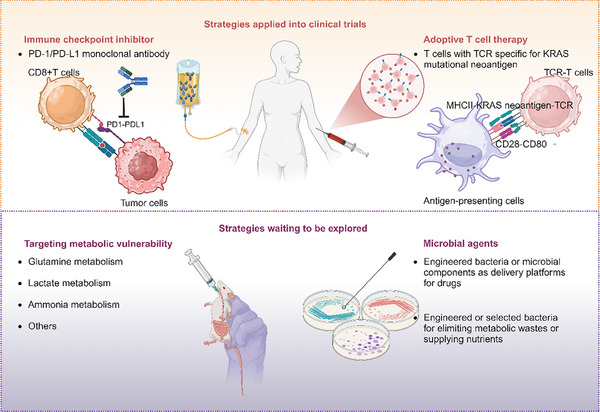
Overview of the tumor microenvironment regulation strategies in KRAS mutant tumors.

As KRAS inhibitors have entered the multi‐KRAS era, their combination with other therapies is expected to be a trend. Since tumors with KRAS mutations were characterized by reduced infiltration and inactivation of CD8+ T cells in the immune microenvironment, the efficacy of KRAS inhibitors relied on the presence and activation of CD8+ T cells for effective tumor elimination.^[^ ^60^, ^61^ ^]^ Therefore, targeting the enhancement of CD8+ T cell function was an important combined therapeutic strategy. PD‐1/PD‐L1 monoclonal antibodies relieved the inhibitory effects of the PD‐1/PD‐L1 signaling pathway on CD8+ T cells, restoring their ability to kill tumor cells. The combination of these antibodies with KRAS inhibitors has shown improved control of tumor compared to monotherapy,^[^ ^11^, ^59^, ^60^ ^]^ and is one of the most promising areas of exploration at this stage, with multiple clinical trials currently underway (**Figure** **7** and **Table** **3**). T cell receptor‐engineered T cells (TCR‐T) engineered to target the new antigens associated with KRAS G12D/V can specifically recognize KRAS G12D/V mutant peptides and directly kill tumor cells by secreting cytokines, granzyme B, and perforin.^[^ ^101^ ^]^ This approach represents a promising immunotherapeutic strategy for treating KRAS mutant solid tumors, and there are also relevant clinical trials underway (Figure 7 and Table 3).

**Table 3:** Clinical Trials of the Combination of KRAS Inhibitors and ICIs or TCR‐T Targeting KRAS.

Targeted drugs	Target	Combined ICI	Tumor type	NCT number	Phase	Sponsor	Status	Refs.
Sotorasib	KRAS G12C	Anti‐PD‐1/L1	Advanced solid tumors	NCT03600883	Phase 1/ 2	Amgen	Active, not recruiting	[16]
Sotorasib	KRAS G12C	Anti‐PD‐1/L1(Pembrolizumab or Atezolizumab)	Advanced solid tumors	NCT04185883	Phase 1	Amgen	Recruiting	[18]
Adagrasib	KRAS G12C	Pembrolizumab	NSCLC ^a)^	NCT04613596	Phase 2/ 3	Mirati Therapeutics Inc.	Recruiting	[118]
Adagrasib	KRAS G12C	Nivolumab	NSCLC	NCT05472623	Phase 2	Sidney Kimmel Comprehensive Cancer Center at Johns Hopkins	Recruiting	/
Adagrasib	KRAS G12C	Pembrolizumab	NSCLC	NCT05609578	Phase 2	Mirati Therapeutics Inc.	Recruiting	/
Adagrasib	KRAS G12C	Pembrolizumab	Advanced solid tumors	NCT03785249	Phase 1/ 2	Mirati Therapeutics Inc.	Recruiting	[17, 33, 119]
Adagrasib	KRAS G12C	Durvalumab	NSCLC or gastro‐intestinal cancer	NCT05848843	Phase 1	M.D. Anderson Cancer Center	Recruiting	/
RMC‐6291/ RMC‐6236	KRAS G12C	Pembrolizumab	NSCLC	NCT06162221	Phase 1/ 2	Revolution Medicines, Inc.	Recruiting	/
Divarasib	KRAS G12C	Pembrolizumab	NSCLC	NCT05789082	Phase 1/ 2	Hoffmann‐La Roche	Recruiting	[120]
JDQ443	KRAS G12C	Tislelizumab	Solid tumors	NCT04699188	Phase 1/ 2	Novartis Pharmaceuticals	Recruiting	[121]
LY3537982	KRAS G12C	Pembrolizumab	Advanced solid tumors	NCT04956640	Phase 1/ 2	Eli Lilly and Company	Recruiting	[122]
MK‐1084	KRAS G12C	Pembrolizumab	Advanced solid tumors	NCT05067283	Phase 1	Merck Sharp & Dohme LLC	Recruiting	[123]
IBI351	KRAS G12C	Sintilimab	NSCLC	NCT05504278	Phase 1	Innovent Biologics (Suzhou) Co. Ltd.	Recruiting	/
GDC‐6036	KRAS G12C	Atezolizumab	Advanced solid tumors	NCT04449874	Phase 1	Genentech, Inc.	Recruiting	[19]
NT‐112 (TCT‐T^b)^)	KRAS G12D	/	Solid tumors	NCT06218914	Phase 1	Neogene Therapeutics, Inc.	Recruiting	[124]
AFNT‐211 (TCT‐T)	KRAS G12V	/	Solid tumors	NCT06105021	Phase 1/ 2	Affini‐T Therapeutics, Inc.	Recruiting	[125]

^a)^ NSCLC, nonsmall cell lung cancer;

^b)^ TCT‐T, T cell receptor‐gene engineered T cells.

This figure analyzed the tumor microenvironment regulation strategies that underwent clinical trials or deserve to be developed in the laboratory. Both immune checkpoint inhibitors and adoptive T cell therapies were the strategies nearest to clinic success. Therapeutic targets based on the metabolic or microbial microenvironment were still in early stages, but deserved to be further explored.

Antiangiogenic agents are a therapeutic approach for cancer. Although KRAS promotes the generation of neovascularization in tumors, there is currently insufficient research evidence to demonstrate a correlation between KRAS mutation status and the efficacy of antiangiogenic agents.^[^ ^102^ ^]^ Therefore, antiangiogenic agents may not be an optimal combination strategy with KRAS inhibitors. KRAS mutations promoted metabolic reprogramming, increasing tumor dependency on glutamine, lactate, and ammonia. Targeting key enzymes or products in these metabolic pathways is a promising research direction. However, the regulatory effects of metabolism on tumor cells and immune responses are complex. Some studies suggested that blocking glutamine metabolism could reprogram T cell metabolism and directly activate T cells.^[^ ^103^ ^]^ Conversely, some studies indicated that inhibiting glutaminase, while suppressing tumor growth, also disrupted the activation and antitumor capacity of dendritic cells and CD8+ T cells.^[^ ^70^, ^104^ ^]^ Given that glutamine is an essential fuel for both tumor and immune cells, a key issue in this field is how to promote glutamine utilization in immune cells while inhibiting its uptake by cancer cells. Lactate and ammonia in the microenvironment are metabolic waste products generated by tumor cells, posing metabolic stress on immune cells. Therefore, inhibiting the sources of these substances or promoting their clearance from the microenvironment is theoretically a feasible therapeutic strategy. Additionally, inhibitors of the lactate transporter MCT4 and agents that clear ammonia from the blood can reactivate T cells and limit tumor growth.^[^ ^69^, ^74^ ^]^

Most of the current KRAS inhibitors did not have targeted delivery systems, which limited their efficacy in tumor targeting.^[^ ^105^ ^]^ Engineered bacteria or microbial components can serve as effective delivery systems due to their tumor targeting property.^[^ ^105^, ^106^ ^]^ Some bacteria can remove metabolic waste from tumors or provide essential nutrients for antitumor immunity. The high‐lactate‐metabolizing photosynthetic bacteria LAB‐1 and engineered probiotic *Escherichia coli* Nissle 1917, described in Section 6, specifically and persistently colonized tumor tissues to eliminate lactate and ammonia from the microenvironment while supplying arginine.^[^ ^99^, ^100^ ^]^ Microbes also modulated tumor sensitivity to immune checkpoint therapies.^[^ ^14d^ ^]^ CRISPR‐engineered *Acinetobacter baylyi* acted as a sensor for KRAS, assisting in the noninvasive diagnosis of KRAS mutations and subsequent treatment decisions.^[^ ^107^ ^]^ Therefore, targeted therapies focusing on microbes and their possible combinations with KRAS inhibitors are worth exploring. However, research on how microbes affect the efficacy of KRAS inhibitors remains sparse. Future studies could focus on microbial populations related to resistance to KRAS mutation inhibitors or microbial formulations for targeted delivery systems, providing novel insights for treating KRAS mutant tumors.

Moreover, KRAS mutations play a role in shaping the TME early in tumor development. A study focusing on PanIN samples demonstrated the early presence of KRAS mutations and their carcinogenic potential in the microenvironment.^[^ ^108^ ^]^ We have also summarized the role of KRAS mutations in PanIN in previous sections. In addition to further research on the early role of KRAS mutations in various cancers, it is a pertinent question whether KRAS inhibitors or precise microenvironmental modulation therapies should be explored, and how opportunities for their application in individuals at the early stages of tumor development can be identified. This may represent a potential avenue for preventive treatment in high‐risk KRAS‐associated cancer patients, though it seems there is still a long and winding road ahead.

## Conflict of Interest

The authors declare no conflict of interest.

## Author Contributions

J.M., and S.F. contributed equally to this work. C.C., Y.P., and S.Z. conceived the project. J.M. and S.F. wrote the manuscript. J.T., Y.H., Y.C., X.D., and H.S. revised the manuscript. All authors read and approved the final manuscript.
